# Thank You to Our Peer Reviewers!

**Published:** 2018-01-01

In this issue of *JADPRO*, we would like to acknowledge everyone who completed peer reviews for *JADPRO* in the past year. Our reviewers, who are from a wide variety of oncology specialties, dedicate their expertise and time to provide insightful feedback on submitted manuscripts.

We hope that during the past year we have fully given you our thanks and made our appreciation to you clear. We hope that publishing the names of all of the *JADPRO* peer reviewers will be another step toward giving you the recognition you deserve for your incredible work. We couldn’t do it without you!

If you’d like to become a peer reviewer for *JADPRO*, please contact ckiffer@hbside.com for more information about how to get involved.

—From the Editors of *JADPRO*

**Sandra Allen-Bard, MSN, ANCC, AOCNP®**

**Paula Anastasia, RN, MN, AOCN®**

**Laurie Appleby, NP**

**Erin Armideo, RN, CRNP, CPON**

**Tyler Beardslee, PharmD**

**Carol Blecher, RN, MS, AOCN®, APNC, CBPN-C, CBCN**

**Catherine S. Bishop, DNP, NP, AOCNP®**

**Leigh M. Boehmer, PharmD, BCOP**

**Tami Borneman, RN, MSN, CMS**

**Jeannine M. Brant, PhD, APRN, AOCN®**

**Kevin Brigle, PhD, ANP**

**Christopher J. Campen, PharmD, BCOP**

**Kimbra Cardwell, MSN, APRN**

**Amanda Cashen, MD**

**Christine Cifranic, PA-C, MPAS**

**Amber Clemmons, PharmD, BCOP**

**Lorinda A. Coombs, PhDc, FNP-BC, AOCNP®**

**Diane G. Cope, PhD, ARNP, BC, AOCNP®**

**Lisa Cordes, PharmD, BCACP, BCOP**

**Marianne Davies, DNP, ACNP, AOCNP®**

**Jessica Davis, FNP, AOCNP®, ACHPN**

**Mary Elizabeth Davis, RN, MSN, AOCNS®**

**Deena Damsky Dell, MSN, RN-BC, AOCN®**

**Morgane Diven, PharmD, BCOP**

**Ly Dsouza, PA-C**

**Raj Duggal, PharmD, BCOP**

**Beth Eaby-Sandy, MSN, CRNP, OCN®**

**Colleen Erb, MSN, ACNP-BC, AOCNP®**

**Enza Esposito-Nguyen, RN, MSN, ANP-BC**

**Beth Faiman, PhD, MSN, APRN-BC, AOCN®**

**Nataya Francis, MS, ARNP, AOCNP®**

**Jessica Gahres, PA-C**

**Elizabeth Gilbert, MS, PA-C**

**Theresa Wicklin Gillespie, PhD, MA, RN**

**Sherry Goldman, NP, RN, CBCN**

**Amy Goodrich, MSN, CRNP-AC**

**Carolyn Grande, CRNP, AOCNP®**

**Heather Greene, MSN, FNP, AOCNP®**

**Kristina M. Gregory, RN, MSN, OCN®**

**Carol Guarneri, RN, MSN, FNP-C, AOCNS®**

**Clell Hasenbank, PharmD**

**Susan Hawbaker, MSN, APN, ANP-BC, OCN®**

**Lisa Henczel, MSN, NP(F)**

**Rhonda Hewitt, MSN, AOCNP®, ANP**

**Brianna Hoffner, MS, ANP-BC, AOCNP®**

**Genevieve Hollis, MSN, CRNP, ANP-BC, AOCN®**

**Patrick Horne, MSN, ARNP, FNP-BC**

**Joyce Jackowski, MS, FNP-BC, AOCNP®**

**Kate D. Jeffers, PharmD, BCOP**

**Joseph A. Kalis, PharmD, BCOP**

**Patricia Karwan, DNP, APRN-BC**

**Soo Jung Kim, MSN, ANP, AGPCNP-BC**

**Kate Kravits, MA, RN, HNB, LPC, NCC, ATR-BC**

**Kristen Kreamer, CRNP, MSN, AOCN®, APRN-BC**

**Sandra E. Kurtin, RN, MS, AOCN®, ANP-C**

**Gail Kwarciany, RN, MSN, BC, OCN®, AOCNS®**

**Lindsey Law, MPAS, PA-C**

**Joanne Lester, PhD, CRNP, ANP-BC, AOCN®**

**Karla Lingard, RNBSc (Hons)**

**Lindsey Lyle, MS, PA-C**

**Steve Malangone, MSN, FNP-C**

**Melissa Martinez, NP**

**Kelley D. Mayden, MSN, FNP, AOCNP®**

**Ali McBride, PharmD, BCOP**

**Erin McMenamin, MSN, CRNP, ANP-BC, AOCN®**

**Gretchen McNally, PhD, ANP-BC, AOCNP®**

**Lynora Metoyer, DNP, FNP**

**Marcia Mickle, ACNP, AOCN®**

**Kim Mullins, DNP, APRN, AOCNP®**

**Susan Newton, RN, MS, AOCN®, AOCNS®**

**Nancy M. Nix, PharmD, BCPS, BCOP**

**Patricia Palmer, RN, MS, AOCNS®**

**Jennifer Paul, PA-C**

**Linda Penwarden, MN, RN, AOCN®**

**Mary E. Peterson, MS, APRN, AOCNP®**

**Mary Jo Pilat, PhD, MS, PA-C, CCRP**

**Julie Ponto, PhD, RN, ACNS-BC, AOCNS®**

**Allyson Price, MPAS, PA-C**

**Carol Proud, MSN, CRNP, AOCNP®**

**Krista Ramirez, PharmD, BCPS**

**Jacqueline Roberts, DNP, FNP-BC, AOCNP®**

**Barbara Rogers, CRNP, MN, AOCN®, ANP-BC**

**Deborah Rust, MSN, CRNP, AOCN®**

**Joanne C. Ryan, PhD, RN**

**Leigh A. Samp, MPAS, PA-C**

**Samantha Schmidt, PharmD**

**Joanna Schwartz, PharmD, BCOP**

**Julie Scott, ANP-BC**

**Gary Shelton, DNP, NP, ANP-BC, AOCNP®, ACHPN**

**Cara Simon, PhD, CPNP**

**Julie A. Spears, APRN-CNS, CCNS, AOCNS®, CNP**

**Mady Stovall, RN, MSN, ANP-BC**

**Sarah Tinsley, PhD, ARNP, AOCN®**

**Alison Holmes Tisch, MSN, RN, ANP-BC, AOCNP®**

**Janice Tran, RN, MS, AOCNP®, CBCN, ANP-C**

**Karley M. Trautman, DNP, ANP-BC**

**Susan Varghese, RN, MSN**

**Jill Vershel, MS, PA-C**

**Carol S. Viele, RN, MS, CNS, OCN®**

**Constance Visovsky, PhD, RN, ACNP-BC**

**Wendy H. Vogel, MSN, FNP, AOCNP®**

**Elizabeth Waxman, RN, MSN, AOCN®, NP-C**

**Steven H. Wei, MS, MPH, PA-C**

**Alyona Weinstein, RN, MSN, FNP-BC**

**Kate Whitelaw, PA-C**

**Rita Wickham, PhD, RN, AOCN®**

**Mali Wold, MSN, NP, RN**

**Tara Wray, RN, MSN, APN-BC**

